# Investigation of Photoexcitation Energy Impact on Electron Mobility in Single Crystalline CdTe

**DOI:** 10.3390/ma14154202

**Published:** 2021-07-28

**Authors:** Viktor Djurberg, Saman Majdi, Nattakarn Suntornwipat, Jan Isberg

**Affiliations:** Division for Electricity, Department of Electrical Engineering, Uppsala University, P.O. Box 65, 75103 Uppsala, Sweden; Saman.Majdi@angstrom.uu.se (S.M.); Nattakarn.Suntornwipat@angstrom.uu.se (N.S.); jan.isberg@angstrom.uu.se (J.I.)

**Keywords:** ToF, time of flight, scattering, drift velocity, CdTe, cadmium telluride, mobility

## Abstract

The exceptional electronic properties of cadmium telluride (CdTe) allow the material to be used in a wide range of high energy radiation detection applications. Understanding the mechanisms of local carrier scattering is of fundamental importance to understand the charge transport in the material. Here, we investigate the effect of photoexcitation on electron transport properties in chlorine doped single crystalline cadmium telluride (SC-CdTe:Cl). For this purpose time of flight measurements were performed on SC-CdTe:Cl in order to study the electron drift mobility in the low injection regime. Measurements were made at the temperature intervals of 80 to 300 K, for an applied electric field between 270 and 1600 V/cm and for wavelengths of 532, 355 and 213 nm. We have found that the electron drift mobility was affected by the excitation energy for temperatures below 200 K. In addition, the measurements revealed that it is possible to determine impurity and shallow trap concentration by this method. The method proves to be extremely sensitive in measuring very low impurity levels and in identifying dominant scattering mechanisms.

## 1. Introduction

Single crystalline cadmium telluride (SC-CdTe) is considered a promising semiconductor material in many applications, e.g., optoelectronics [1,2], radiation detectors [3,4] and Gunn oscillators [5]. The material exhibits a high mobility lifetime (*μτ*) product, which is important in order to achieve high spatial resolution and high counting efficiency in detector applications. Due to the rapid improvement in growth technologies, particularly the traveling heater method (THM) [6,7], it is now possible to achieve high resistivity chlorine doped single crystalline cadmium telluride (SC-CdTe:Cl) substrates. In CdTe:Cl, charge carrier mobility tends to be limited by defect scattering at low temperatures [8]; hence, a low impurity concentration is important. At higher temperatures, polar optical phonon scattering tends to be the dominating mechanism [9,10]. 

Usually, Hall effect measurements are used to investigate the mobility of charge carriers in semiconductors. However, this technique cannot be applied in the case of insulating or high resistivity CdTe:Cl as it gives indistinct results due to mixed electron and hole conduction. Instead, the time of flight (ToF) method, also known as the transient current technique (TCT), can be applied. In this case, electron–hole pairs are created by *α*-particles [11,12], pulsed electron beams [13,14], pulsed x-rays [15], or a pulsed laser [16,17,18,19,20] with specific excitation energies. In the ToF experiment, the motion of the free charge carriers in an applied electric field induces a current which is then measured. Depending on the applied bias polarity, conduction dominated by electrons or holes can be investigated separately. In addition, the system enables drift velocity $\;(v_{d})$ and mobility measurements in a wide temperature range and it is possible to determine defect concentrations. 

Even though charge transport studies have been previously performed on CdTe, the impact of photoexcitation energy on transport characteristics has never been investigated. Therefore, we investigate the transport properties in SC-CdTe:Cl at different photoexcitation energies and as a function of temperature. By employing the ToF technique, we were able to measure the drift velocity and the mobility of electrons in the temperature range of 80 to 300 K at applied electric fields of 270 to 1600 V/cm. The experimental observations reveal an increase in mobility with decreasing temperatures down to 100 K. At temperatures below 100 K, a plateau followed by a decrease in the electron mobility is observed in all samples. This behavior can be attributed to the presence of defects acting as shallow traps and causing ionized impurity scattering. Any hole transit across the investigated samples could not be observed due to strong trapping. A detailed understanding of key parameters such as the trapping rate of carriers, mobility, and charge collection efficiency are essential for many applications, e.g., radiation detectors. 

## 2. Materials and Methods

**Figure 1:**
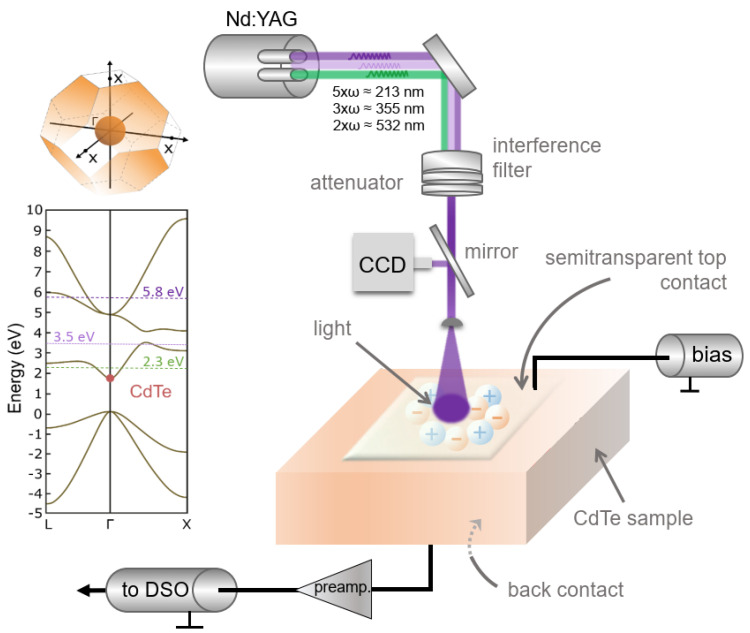
Schematics of the time of flight system used for the measurements. By changing the electric field polarity, it is possible to measure electron and hole drift velocity separately. The conduction band structure and the first Brillouin zone of CdTe are depicted to the left. The dashed and dotted lines in the band diagram correspond to different optical excitation energies provided by different harmonics of the YAG-laser.

A set of freestanding, commercially available SC-CdTe:Cl samples, grown in the (111) crystallographic orientation using the THM technique, by Acrorad Co, under conditions of high purity, were studied. Two samples, sample #1 and sample #2, with the dimensions 4 × 4 mm and with a thickness of 1000 µm, were mechano-chemically polished followed by metallization (Ohmic Pt (50 nm)) on both side using electroplating by the manufacturer. Semitransparent mesh contacts (Ø3 mm) were patterned in our lab by photolithography followed by Ar-ion milling on the two opposite faces of the samples. The contact geometry makes it possible to apply a homogenous electric field while illuminating the sample surfaces. The ToF technique was used for measuring the drift velocity at very low carrier concentrations [21,22]. For this purpose, we used a low noise broadband current Mini Circuits 2x ZFL-1000LN+ amplifier (1 GHz bandwidth, 24 dB amplification) together with a Tektronix TDS 684C digital storage oscilloscope (DSO). Bias was applied in 50 µs pulses via a bias-tee. The short pulsed bias ensures capacitive voltage distribution across the sample and avoids undesirable sample polarization. However, the pulse length is several orders of magnitude longer than the transit time of the carriers. Figure 1 illustrates the schematics of the ToF system. 

In the experiment, electron-hole pairs were created by external means using short laser pulses from three different harmonics from an Nd-YAG laser (CryLas GmbH, Berlin, Germany)with wavelengths of 532, 355 and 213 nm (all with higher photon energies than the CdTe band gap of ~1.5 eV). The penetration lengths are below 0.2 µm for all three wavelengths [23]. The intensity can be adjusted using a variable neutral density filter (Thorlabs NDC-50C-2M). Unwanted harmonics were blocked using interference filters. As light is absorbed in the sample, electron-hole pairs are generated close to the illuminated sample surface and it is possible to transport either holes or electrons (depending on the applied bias polarity) through the sample and extract them at a receiving electrode. The induced current, which is related to the carrier motion according to the Shockley-Ramo theorem [24,25], was measured at the receiving electrode by an external circuit. The ToF setup and data evaluation we used is described in more detail in [17,19]. Here, the samples were mounted in a ceramic chip carrier, wire bonded, and placed in a liquid nitrogen cooled Janis ST-300MS vacuum cryostat with UV optical access. The temperature was monitored using a LakeShore 331 temperature controller with a calibrated TG-120-CU-HT-1.4H GaAlAs diode sensor in good thermal contact with the sample.

## 3. Results and Discussion

**Figure 2:**
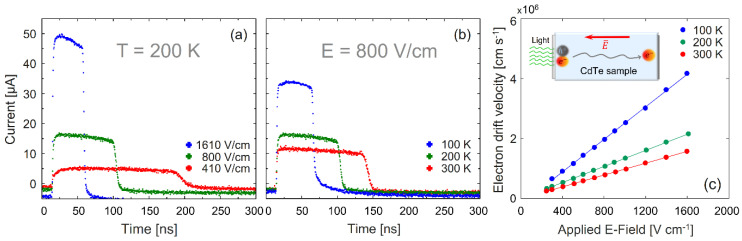
Examples of current traces measured by the ToF technique with 213 nm excitation in sample #1 for (**a**) different electric fields at 200 K and (**b**) different temperatures at an electric field of 800 V/cm. The measured electron drift velocity for different bias field strengths and different temperatures are presented in (**c**). The inset illustrates charge generated by the photoexcitation and the carrier drift through the sample.

Typical electron current waveforms obtained with 213 nm photoexcitation are plotted in Figure 2 for sample #1. The measured current traces are shown for varied applied electric fields at 200 K in Figure 2a and for three different temperatures at 800 V/cm in Figure 2b. The clear square shape of the current traces indicates a high quality, low trapped charge, and good homogeneity of the sample. Figure 2a shows the expected decrease in transit time from 180 ns at 410 V/cm to 45 ns at 1610 V/m. As can be seen (Figure 2b), current traces vary from 130 ns at RT to 50 ns at 100 K, showing the increase in the carrier drift velocity at lower temperatures in the 300–100 K temperature region. By fitting error-functions to the rising and falling edges of the current pulse, as described in [19], the transit time is determined. The electron drift velocity $v_{d}$ is calculated as the quotient of the sample thickness d and the transit time  τ. The transit time was measured for three SC-CdTe:Cl samples at temperatures in the range of 80 to 300 K and for applied electric fields in the interval of 270 to 1600 V/cm. For the entire temperature range, a simple linear $v_{d}=\mu E\;$ relation, where μ is the low-field drift mobility and *E* is the electric field strength, is observed (Figure 2c). From the linear behavior, it is clear that carrier heating caused by the applied electric field is negligible even for the highest applied fields. This is the same linear behavior and mobility as has been shown for pure CdTe at RT, and a factor 2 higher compared to the mobility of CdS and ZnS [9,26]. However, at 100 K, the mobility is a factor 5 lower Cl:CdTe compared to pure CdTe [9]. 

**Figure 3:**
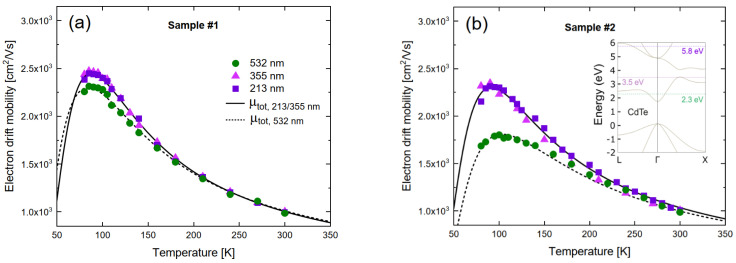
(**a**,**b**) Measured mobility for sample #1 and #2 as function of temperature for three different excitation wavelengths respectively. The solid lines represent the fitted mobility including the effect of shallow traps and scattering. Inset in (**b**): Energy band diagram for CdTe indicating the provided laser energies by dotted and dashed lines.

For temperatures below 200 K, the photoexcitation energy affects the measured mobilities, as can be seen in Figure 3. The measurement with the 532 nm laser yields a lower mobility compared to 355 and 213 nm illumination. Since the relaxation time of the excited electrons is much shorter than the transit time, this phenomenon is related to the photoexcitation at the surface. First we discuss the possibility of a resident space charge in the bulk of the sample that could affect the electric field distribution and, consequently, the drift velocity of the electrons. However, it turns out that the possibility of a bulk charge large enough to affect the measured drift mobility can be excluded by analyzing the current traces in detail [27]. Another possible explanation is that there is charge trapped at the surface that affects the electric field throughout the sample. In fact, the observed behavior is consistent with a trapped positive surface charge in the case of 532 nm photoexcitation, which does not manifest itself for the shorter wavelengths. It is clear that further investigation of this phenomenon is required in order to ascertain the origin, but, irrespective of its source, it is important to take this effect into account when comparing data using different excitation sources. In the following, we will use the 355 and 213 nm photoexcitation data, as these are in agreement, to determine trap densities.

The measured mobility for both high and low excitation energy can be well described with a model including contributions from shallow traps, ionized impurity scattering and polar optical phonon scattering [28]. Piezoelectric, neutral impurity and deformation potential scattering have been excluded from the model because they have been shown to be at least two orders of magnitude smaller compared to polar optical phonon scattering [8]. The mobility $\mu_{tot}$ is given by the expression:

(1) $$\mu_{tot}=\mu_{0}\left(T\right){\left[1+\frac{N_{t}}{N_{c}}e^{\frac{E_{t}}{K_{b}T}}\right]}^{-1}\;$$

where $N_{t}$ and $\;E_{t}$ are the shallow trap density and energy, respectively, $N_{c}$ is the effective conduction band density of states and $\mu_{0}$ is a combined mobility, using Matthiessen’s rule, resulting from ionized impurity and polar optical scattering. In our analysis, $E_{t}$ was set to 14 meV, which corresponds to the Cl donor level in CdTe:Cl [29].

The contribution to the combined mobility from ionized impurity scattering can best be described with a truncated coulomb potential model (Cornwell and Weisskopf (CW)) [30]. The mobility can be extracted from the CW model:

(2) $$\mu_{CW}=\frac{128\sqrt{2\pi \;}\epsilon_{0}^{2}\epsilon_{r}^{2}{\left(k_{b}T\right)}^{\frac{3}{2}}}{\sqrt{m_{\mathrm{eff}}}N_{i}Z^{2}e^{3}}{\left[\ln\left(1+{\left(\frac{12\pi \epsilon_{0}\epsilon_{r}k_{b}T}{\sqrt[{3}]{N_{i}}Ze^{2}}\right)}^{2}\right)\right]}^{-1}$$

where *N_i_* is the ionized impurity concentration, *Z* is the charge of the impurity, $m_{\mathrm{eff}}$ is the effective mass of the charge carrier and e is the element charge. The polar optical mobility for a nondegenerate semiconductor with one band was calculated by Ehrenreich [31]. Using the Callen charge [32] in order to describe the effective ionic charge, the mobility can be reduced to:

(3) $$\mu_{pop}=\frac{0.870}{\alpha k_{b}\theta }\frac{m}{m_{\mathrm{eff}}}\frac{e^{z}-1}{{\left(z\right)}^{\frac{1}{2}}}\;G\left(z\right)e^{\xi }$$

where, *z* = θ/*T*, where θ is the Debye temperature and α is the Fröhlich coupling constant [33], which describes the strength of the interaction between electrons and longitudinal optical phonons in a polar semiconductor. $G\left(z\right)e^{\xi }$ is a function that Howarth and Sondheimer [34] developed to include the interactions of charge carriers. For low charge carrier concentrations, $e^{\xi }=1$ and G(z) is a slowly varying function close to 1.

**Figure 4:**
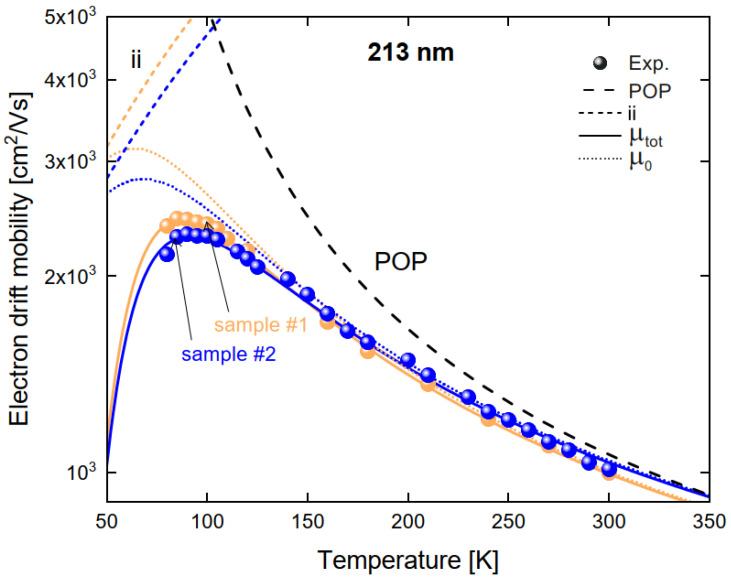
The mobility as function of temperature for two SC-CdTe:Cl samples measured by the ToF technique with 213 nm illumination. The dashed and broken lines represent the mobilities from ionized impurity (ii) and polar optical phonon scattering (POP), separately. Solid lines represent the fitted mobility including the effect of shallow traps and scattering, while the dotted line is the mobility only considering ionized impurity and polar optical phonon scattering.

By least squares fitting the mobility expression given in Equation (1) to the measured mobilities from the 355 and 213 nm lasers, it is possible to extract the ionized impurity, shallow trap concentrations and the Fröhlich coupling constant. Figure 4 shows the experimental mobilities together with a weighted nonlinear least square fit. For the Fröhlich coupling constant we obtained the best fit for α = 0.53 ± 0.02. The ionized impurity and shallow trap concentrations obtained are summarized in Table 1. It is important to note that our model is based on Matthiessen’s rule, which tends to underestimate the scattering for different sources in compound semiconductors by around 10–20% [35,36], though cases of around 60 % have been shown in extreme cases [37]. Considering this, the shallow trap concentrations in Table 1 can be interpreted as upper limits for these concentrations. The ionized impurity concentration is lower (indicating the improvement of the crystal quality) than in previous studies where Matthiessen’s rule was used [38].

## 4. Conclusions

In summary, we have measured the electron mobility for a wide range of temperatures and with three different photoexcitation energies in SC-CdTe:Cl using a time of flight method. We discovered that the laser photoexcitation energy has an impact on the measured mobility which was attributed to the build up of a surface charge for the longest excitation wavelength. The measured mobilities were in agreement with the presented model and previous data. This shows that the dominating scattering mechanics are ionized defect scattering at low temperatures and polar optical scattering at high temperatures. It was also possible to measure ionized impurity concentrations in the samples, which are of the order of 10^16^ to 10^17^ cm^−3^, which are the lowest reported, to our knowledge, and is an important parameter for the low temperature performance of SC-CdTe:Cl detectors. We have also measured an upper limit for the shallow trap concentration of 10^15^ cm^−3^ which, along with trap energy, affects the lowest possible temperature at which the detector can be used.

## Figures and Tables

**Table 1:** Extracted parameters for impurity concentration ***N_i_*** and shallow trap concentration ***N_t_*** for 532 and 213 nm photoexcitation. The confidence interval is calculated from the least-squares fit.

Sample	*N_i_* × 10^16^ [cm^−3^]	*N_t_* × 10^14^ [cm^−3^]
*# 1*	6.4 ± 0.4	60 ± 12
*# 2*	7.7 ± 0.8	55 ± 18

## Data Availability

The data that support the findings in this study are available from the corresponding author upon reasonable request.
